# Ancestral alleles defined for 70 million cattle variants using a population-based likelihood ratio test

**DOI:** 10.1186/s12711-024-00879-6

**Published:** 2024-02-06

**Authors:** Jigme Dorji, Antonio Reverter, Pamela A. Alexandre, Amanda J. Chamberlain, Christy J. Vander-Jagt, James Kijas, Laercio R. Porto-Neto

**Affiliations:** 1 CSIRO, Agriculture & Food, St. Lucia, QLD 4067 Australia; 2 AgriBio, Centre for AgriBioscience, Agriculture Victoria, Bundoora, VIC 3083 Australia

## Abstract

**Background:**

The study of ancestral alleles provides insights into the evolutionary history, selection, and genetic structures of a population. In cattle, ancestral alleles are widely used in genetic analyses, including the detection of signatures of selection, determination of breed ancestry, and identification of admixture. Having a comprehensive list of ancestral alleles is expected to improve the accuracy of these genetic analyses. However, the list of ancestral alleles in cattle, especially at the whole genome sequence level, is far from complete. In fact, the current largest list of ancestral alleles (~ 42 million) represents less than 28% of the total number of detected variants in cattle. To address this issue and develop a genomic resource for evolutionary studies, we determined ancestral alleles in cattle by comparing prior derived whole-genome sequence variants to an out-species group using a population-based likelihood ratio test.

**Results:**

Our study determined and makes available the largest list of ancestral alleles in cattle to date (70.1 million) and includes 2.3 million on the X chromosome. There was high concordance (97.6%) of the determined ancestral alleles with those from previous studies when only high-probability ancestral alleles were considered (29.8 million positions) and another 23.5 million high-confidence ancestral alleles were novel, expanding the available reference list to improve the accuracies of genetic analyses involving ancestral alleles. The high concordance of the results with previous studies implies that our approach using genomic sequence variants and a likelihood ratio test to determine ancestral alleles is appropriate.

**Conclusions:**

Considering the high concordance of ancestral alleles across studies, the ancestral alleles determined in this study including those not previously listed, particularly those with high-probability estimates, may be used for further genetic analyses with reasonable accuracy. Our approach that used predetermined variants in species and the likelihood ratio test to determine ancestral alleles is applicable to other species for which sequence level genotypes are available.

**Supplementary Information:**

The online version contains supplementary material available at 10.1186/s12711-024-00879-6.

## Background

Ancestral alleles are the allelic state of the last common ancestor of a group of organisms or, in other words, the alleles that have retained their initial state. They are determined by comparing the genomic sequence of different populations and identifying alleles that are shared by closely-related species or populations [1–3]. Ancestral alleles provide valuable information about the evolutionary history of a particular group of genes or organisms [4], and they are useful to construct phylogenetic trees, study the genetic diversity and structure of populations, infer the demographic history of populations [5–7], identify functional elements in non-coding regions of the genome, and understand the genetic susceptibility to diseases and deleterious alleles [8, 9].

In cattle, ancestral alleles have been determined by several studies [10–12] and used for studies on signatures of selection of traits associated with adaptation and production [13–16]. These ancestral alleles were based on the previous version of the bovine genome (e.g., UMD3.1 or UMD3.1.1) and are focused on the 50k and high-density single nucleotide polymorphism (HD SNP) chip genotypes (e.g. BovineHD and BovineSNP50). In the absence of a comprehensive list of ancestral alleles, an alternate practice is to use major or common alleles as the ancestral alleles [17–19], but it is known that the major alleles are not always the ancestral ones. For example, up to 19% of the identified ancestral alleles in previous studies were minor alleles [10, 20]. Thus, the direct use of the major alleles as the ancestral alleles can potentially jeopardise inferences in genetic studies. More recently, lists of whole-genome sequence-based ancestral alleles (up to 42 million) have been determined in cattle [20, 21]. While this is a significant leap in the number of ancestral alleles determined, it represents less than 28% of the total number of variants detected in cattle (152 million, Run9, the 1000 Bull Genomes project [22]) and a considerable gap still exists at the sequence level.

Generally, ancestral alleles in cattle have been determined by comparing the alleles present within a range of the evolutionarily diverged out-species group of non-cattle *Bos* species and non-*Bos* lineages. In practice, this approach is complex as it needs to consider which out-species should be chosen, and which analytical approaches and threshold should be used to call ancestral alleles. For example, Xiang et al. [20] used three out-species (yak, sheep and camel) and determined probabilities of allele ancestrality using a likelihood ratio test [23], while Naji et al. [21] used four non-cattle *Bos* species (gayal, gaur, yak and banteng) and bison as the out-species group. Rocha et al. [10] conducted comparative analyses of the cattle reference genome to reference genomes of sheep (*Ovis aries*), water buffalo and yak (*Bos grunniens*) and then annotated the SNPs to determine ancestral alleles. Considering the number and diversity of cattle, the use of more diverse cattle breeds in Rocha et al. [10] yielded more variants within cattle and resulted in a longer list of ancestral alleles compared to studies involving fewer breeds [20, 21]. There is also some variation in the threshold adopted to call an allele ancestral, based either on the prevalence of an allele in two-thirds of the species/groups [10, 21] or the use of probability estimates [20]. The combination of the two approaches would enable to define ancestral alleles with higher confidence. Taken together these results highlight the need to capture the maximum variation within cattle and the out-species group for a better coverage of the genome and to assign probability estimates or reliability scores for ancestral alleles.

Currently, the 1000 Bull Genomes project [22] is the largest sequence repository of bovine genomic variation representing most of the major breeds, crossbreeds, and composites across the globe, primarily for imputation and genome-wide association studies. The list of variants in this project may be directly explored for the determination of ancestral alleles in cattle. Additional non-cattle *Bos* species (*B. sauveli*) sequences have recently become available [24] and can also be part of the out-species group. In addition, sequences of another non-cattle *Bos* species (*B. mutus*), which is the wild type of the domestic yak, have not been used previously for the determination of ancestral alleles in cattle. These available resources, together with the use of appropriate statistical methods, are expected to further improve ancestral allele coverage as well as the sensitivity of ancestral allele determination and, hence, the power in genetic analyses associated with ancestral alleles in cattle. Lastly, statistical approaches to estimate the confidence and reliability of ancestral alleles (i.e., probability estimates and the number of out-species supporting ancestrality) would enable the user to make informed decisions about the confidence level of the ancestral alleles they choose to use in their studies.

Therefore, the aim of our study was to determine ancestral alleles in cattle using a large, predetermined dataset of variants in cattle, an expanded list of out-species and a likelihood ratio test for improved ancestral allele coverage on the cattle genome.

## Methods

### Ancestral allele positions in cattle

This study used genomic variants (SNPs) from run9 of the 1000 Bull Genomes project, derived from over 6000 genomes that cover the major taurine, indicine and composite cattle breeds across the globe. The variants were filtered using variant recalibration with the Genome Analysis Toolkit (GATK) and QD, MQ, MQRankSum, ReadPosRankSum, FS and SOR annotations for SNPs (GATK commands and thresholds are available from the 1000 Bull Genomes project on request). Specifically, the SNPs that passed all the filters (i.e., “PASS”, 41.67 million) and those in the truth sensitivity tranche level for the SNP model at VQS Lod: 0.7381 <  = x < 9.3379 (“VQSRTrancheSNP90.00to99.00”, 41.72 million) from the autosomes were considered to further expand the list (see Additional file 1: Table S1). Furthermore, from these categories keeping only the positions that were biallelic (i.e., removing multiallelic sites) made 67.77 million biallelic variants available for the determination of ancestral alleles. Similarly, 2.36 million biallelic positions were available for analysis on chromosome X.

### Out-species group

**Fig. 1:**
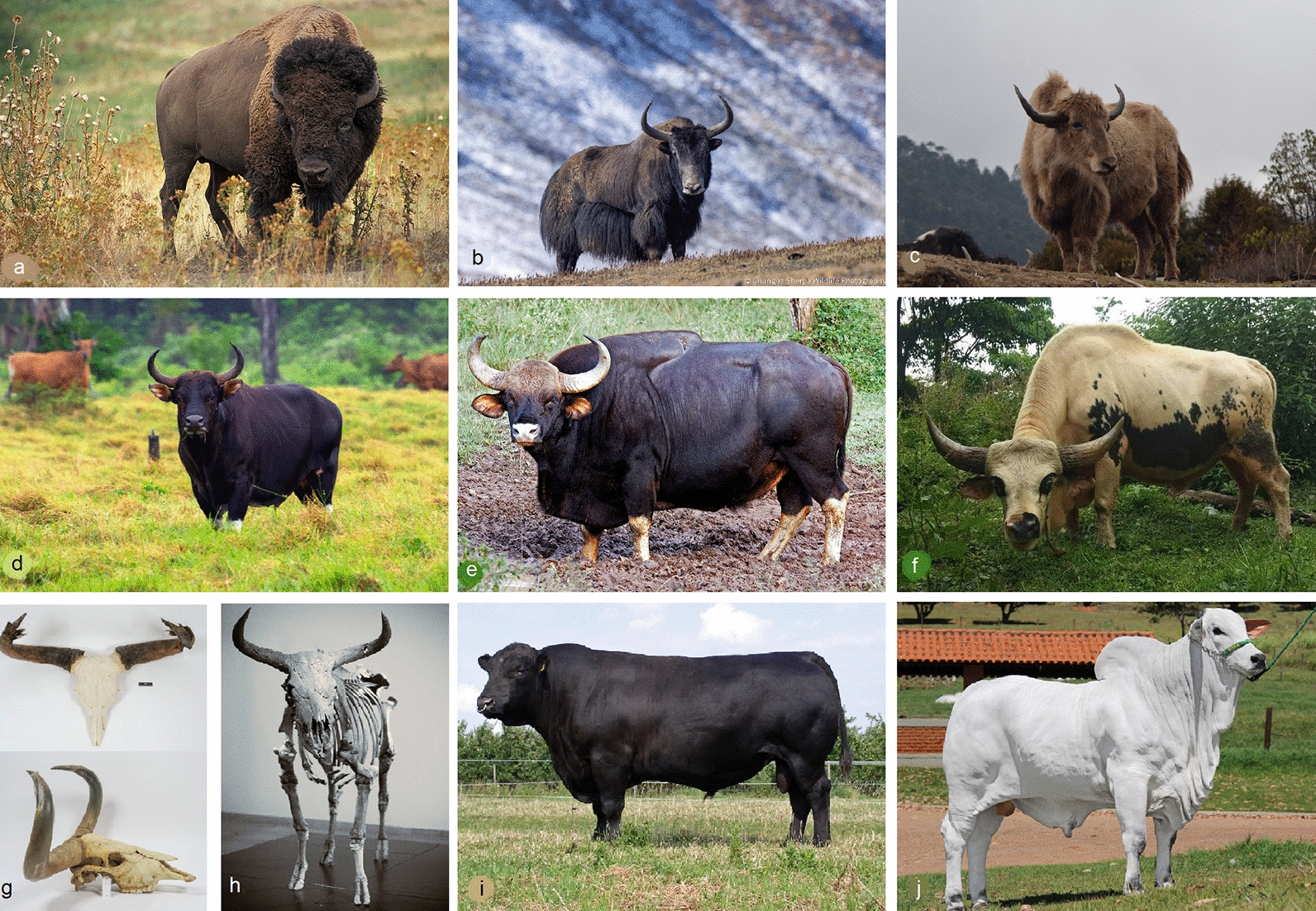
Representative image of the species from out-species and cattle groups sampled in the current study. **a** Bison (*Bison bison*), **b** Wild yak (*Bos mutus*), **c** Domestic yak (*Bos grunniens*). **d** Banteng (*Bos javanicus*), **e** Gaur (*Bos gaurus*), **f** Gayal (*Bos frontalis*), **g** Skull of Kouprey (*Bos sauveli*), **h** Mounted skeleton of Auroch (*Bos primigenius*) bull, **i** Angus bull (*Bos taurus*). **j** Nelore bull (*Bos indicus*) (see Additional file 1: Table S3 for image attributions)

The bovine SNP positions were compared with orthologous positions in an out-species group. The out-species group comprised six non-cattle *Bos* species, including two species previously not used in the determination of ancestral alleles (*Bos sauveli* and* Bos mutus*) and bison as non-Bos out-species (Table 1 and Fig. 1). The wild yaks, in spite of their very subtle phenotypic differences with the domestic yaks, are technically classified as a different species [25], and as such, have never been considered in previous studies that aimed at determining ancestral alleles, and thus were of special interest in this study. Among these species, aurochs (*Bos primegenius*) is listed as extinct and Kouprey (*Bos sauveli*) is critically endangered with a population of less than 50 individuals [26, 27]. While these species are evolutionary divergent and genetically diverse, some closely-related species can inter-breed to produce hybrids [28, 29].

**Table 1:** Summary of the out-species samples processed for the determination of ancestral alleles

Sub-group	Common name	Species	Number of samples
Non-cattle (*Bos* species)	Gayal	*Bos frontalis*	14
	Gaur	*Bos gaurus*	2
	Domestic yak	*Bos grunniens*	11
	Banteng	*Bos javanicus*	9
	Wild yak	*Bos mutus*	4
	Kouprey	*Bos sauveli*	2
Non-*Bos* species	Bison	*Bison bison*	10

See Additional file 1: Table S2 for accession numbers of the samples and associated project

### Analyses

#### Raw sequence read processing

The raw paired-end sequence reads for samples in the out-species group were processed following the guidelines from the 1000 Bull Genomes project (see Additional file 2: Method S1 for tools, command lines and thresholds) for compatibility and comparison with variant positions in cattle from the 1000 Bull Genomes. Similarly, the single-end reads for the *Bos sauveli* samples were aligned and trimmed as appropriate for single-end reads using the same tools as for the paired-end reads and then processed following the same guidelines. We used an updated version of GATK (4.2.0.0) compared to the one mentioned in the guidelines (3.8). The end-product of the pipeline was gVCF files of individual samples, which were then consolidated into a single gVCF file for the out-species group using the GATK CombineGVCFs option. The consolidated gVCF file consisted of 71.35 million positions corresponding to biallelic variants in cattle that were all genotyped, including the non-variant sites (adding option -*include-non-variant-sites true*) in the out-species using joint genotype options (GenotypeVCF) in GATK. After the removal of positions that were multi-allelic and missing in the out-species for the corresponding biallelic positions in cattle, a comprehensive list of 68,256,797 (autosomal) and 2,329,918 (X chromosome) biallelic sites was generated. Note that the X chromosome was not treated differently from the autosomes which means that the sex and hemizygosity of the samples were not considered. Finally, genotypes were conservatively filtered based on read depth (DP) and allele depth (AD) using the BCFtools package [30], i.e. homozygous genotypes with a DP < 3 and heterozygous genotypes with a DP < 5 and any individuals with an AD < 2 were set to missing as a balanced approach between not excessively removing information and tolerating some false positives to take the poor quality of some of the samples into account. Furthermore, filters based on maximum read depth and/or excess heterozygosity call were not applied.

#### Population structure

A preliminary quality check was performed to verify that the processing pipelines had worked by examining the population structure of the out-species group in relation to the cattle group. For cattle, we used a subset of 11 samples from the 1000 Bull Genomes, considering their availability in public databases. This includes the five samples of Nelore cattle (*B. indicus*), another random five Angus samples (*B. taurus*) and one auroch (*B. primigenius*) to constitute the cattle group for the principal component analysis (PCA) (see Additional file 1: Table S2). A subset of the VCF file for the PCA cattle group, specifically for the 70 million genomic positions, was derived from the 1000 Bull Genomes and then merged with the VCF files generated in this study for the out-species using BCFtools (version 1.18.0), resulting in a combined VCF file of cattle and out-species groups. This combined cattle and out-species VCF file was then used for the principal component (PC) analysis, excluding variants with missing call rates higher than 0.1 using the PLINK (version 2.0.0a3.02) software [31]. Furthermore, for a balanced representation of the out-species with only two samples (*B. sauveli* and *B. gaurus*), a relationship matrix was derived using two random samples in the rest of the out-species. The PC and relationship matrices were plotted in the ggplot2 package (version 3.4.2) in R (version 4.0.5) [32]. This was followed by PC and relationship analyses for the out-species group only to assess the structure among the out-species.

#### Determination of ancestral alleles

We used a likelihood ratio test (LRT) to determine the ancestral state of the two alleles at any position on chromosomes 1 to 29 and X (see Additional file 3: Method S2, Additional file 3: Tables S4 to S7 for details on the method with further easy-to-follow examples, Additional file 4: Method S3 for Bash scripts). Briefly, the numerical algorithm proceeds as follows:

1. Based on the genotype frequency at a position in a out-species, the genotype configuration (GT_c_) is derived for that position as the number of samples with A_1_A_1_, A_1_A_2_ and A_2_A_2_ genotypes. For example, on the one hand, in an out-species of 10 samples, if all samples have been genotyped at a position and the number of samples for each genotype A_1_A_1_, A_1_A_2_ and A_2_A_2_ (GT_f_) is 4, 2, 4, then the GT_c_ is 4 2 4. On the other hand, if only seven samples have genotypes (missing in three samples) and have frequencies of 4, 1, 2, the GT_c_ is 4 1 2. Similarly, if all samples have genotypes missing at a position, then GT_f_ and GT_c_ will be 0, 0, 0 and 0 0 0, respectively.
2. Once GT_c_ is determined for all the positions, the number of distinct GT_c_ observed in an out-species (GT_c_obs_) is recorded, and then the number of GT_c_ expected by chance (GT_c_exp_) is determined with an equal probability for all GT_c_.
3. The likelihood ratio (LR) of one allele to be ancestral is calculated as the ratio of GT_c_obs_ to GT_c_exp_ in the out-species and assigned to the allele (A_1_ or A_2_) corresponding to the homozygous genotype with the larger number of samples. For example, let the LR for A_1_ and A_2_ be 0.7 and 1.05, respectively.
4. At the site, a signal for ancestrality is directed to either of the two alleles, with signals adding up to 1 (or 100%). In the above example, the signal for ancestrality would be 0.7/(0.7 + 1.05) or 40% for A_1_ and 1.05/(0.7 + 1.05) or 60% for A_2_.
5. The above steps are repeated for the remaining species in the out-species group.
6. The signals for A_1_ and A_2_ are summed to get the combined signal at a given position for all the out-species group.
7. Finally, the allele with the highest signal is assigned as the ancestral allele and the probability of an allele being ancestral is determined as the proportion of the individual LR to the sum of the LR of the two alleles.
8. In addition to the probability estimate as a measure of confidence of the allele ancestrality, we determined the number of out-species contributing to the probability estimates of the ancestral allele as added weight on the confidence of ancestral alleles. This is because a high probability estimate with representation from all species is potentially more accurate compared to the high probability estimate based on a few out-species. Furthermore, to characterise the positions with a high confidence and the remaining ancestral alleles, we annotated the positions using the snpeff/5.0e tool [33] for any specificity to a region on the genome.

#### Validation of ancestral allele assignments

Ancestral allele assignments generated in this study were compared against three previous studies with comparable numbers of determined ancestral alleles: Rocha et al. [10], Naji et al. [21] and Xiang et al. [20]. The first study was based on the previous bovine reference genome version (UMD3.1), and the latter two used the same reference genome as the present study [20, 21]. For compatibility and comparison, positions from the first study were lifted over from UMD3.1 to ARS-UCD1.2 using the LiftOver tool [34]. Any variants that were mapped to multiple positions following the liftover were removed, considering that such a conversion of the positions is not perfect but is the best guess. Ignoring the strand switches during the liftover process has the potential to introduce errors in the concordance metric after liftover to the newer genome. Nevertheless, it is worth noting that this approach was only applied to the Rocha et al. [10] dataset. For validation of ancestral alleles, we considered only the ancestral alleles with a probability ≥ 0.8 and that were observed in at least six of the seven out-species included in the present study, which we hereafter refer to as high-confidence ancestral alleles. Similarly, ancestral alleles from previous studies were restricted to an equivalent confidence if estimates of probability were provided (probability ≥ 0.8 in the third study) or to all positions if no confidence estimates were provided (first and second study). We determined the number of positions in common between our study and previous studies, and then the percentage agreement of ancestral allele assignments. The overall concordance of ancestral alleles across studies was expressed as the weighted percentage of coincident ancestral alleles, which is expressed as the total number of positions in common among the studies with matching ancestral alleles over the total number of positions in common among the studies. However, owing to X-chromosome ancestral alleles being either unavailable or present in very small numbers in previous studies, the concordance of the ancestral alleles on the X chromosomes were analysed separately from the autosomes, applying the same approach.

## Results

### Population structure

**Fig. 2:**
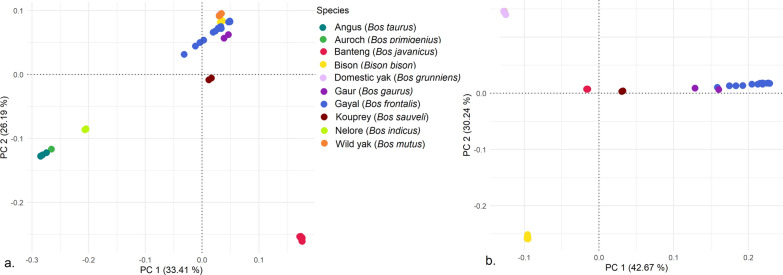
Principal component plot (PC1 and PC2) of cattle and out-species groups (**a**) and within the out-species group (**b**)

**Fig. 3:**
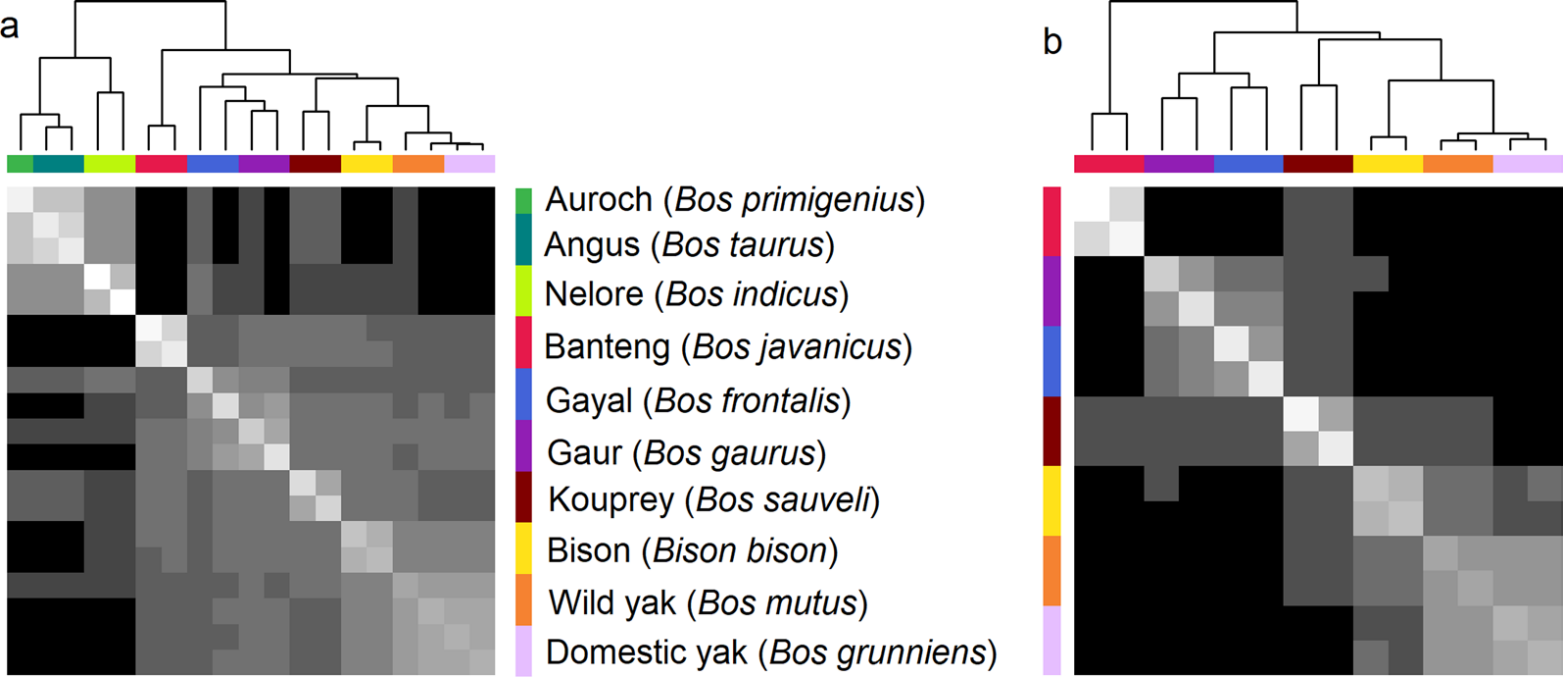
Heatmap of the relationship matrix based on two animals per species for out-species and cattle group (**a**) and out-species only group (**b**)

As a rapid check of the alignment of sequences from the out-species group to the bovine reference genome and the SNP pipeline, PCA were performed for both the cattle and out-species groups. PC1 segregated cattle and their Auroch ancestor from the out-species group (Fig. 2a). Plotting only the out-species group (Fig. 2b) provided a higher resolution of the clusters among the out-species, with PC1 separating *B. javanicus* from the rest of the out-species and PC2 resulting in subgroups for *B. grunniens*, *B. mutus* and *Bison bison*, and for *B. frontalis* and *B. gaurus*. Similarly, the relationship matrix based on the two random animals per species-based relationship matrix (Fig. 3) concurred with the pattern from the PC analyses.

### Ancestral alleles

This study proposed a LRT to determine the ancestral alleles for 67,767,982 biallelic SNP positions in cattle. It should be noted that the designated ancestral allele for 1964 positions remained undetermined either because of heterozygosity or equal signals, and thus removed, which left 67,766,018 sites with determined ancestral alleles (available at https://doi.org/10.25919/9a81-4p83). Via this link, one has access to information on the chromosome number, position (bp), allele 1 (A_1_), allele 2 (A_2_), likelihood ratio (LR) for A_1_ as LRa(A_1_) and A2 as LRa(A_2_), the putative ancestral allele (AA), probability estimate of ancestrality for A_1_ (prob(A_1_)) and A_2_ (prob(A_2_), number of out-species supporting A_1_, number of out-species supporting A_2_, number of out-species supporting AA, and the weight of the out-species support for AA and variant class from the 1000 Bull Genomes. Overall, the mean LR and number of out-species supporting the ancestral allele were high (N 67,766,018, LR: mean 0.983, SD 0.067, min 0.5 and max 1.000; number of out-species supporting ancestral alleles: mean 6.223, SD 0.630; Min 1 and Max 7; see Additional file 5: Table S8).

To further improve the confidence of ancestral alleles, we considered ancestral alleles with an LR ≥ 0.8, which removed 2,127,228 positions and left 65,644,102 positions. In addition, using the number of out-species supporting the ancestral call as another measure of reliability (i.e., the probability estimates of ancestral allele call supported by at least six out-species of the seven in the out-species group) resulted in 61,987,061 high-confidence ancestral alleles. For the X chromosome, ancestral alleles were determined on 2.3 million positions (available at https://doi.org/10.25919/9a81-4p83), and about 91.2% were in the high-confidence category. We also report that around 12.5% of the autosomal and about 10% of the X chromosomal ancestral alleles were represented by minor alleles in the 1000 Bull Genomes project (Table 2). The annotation of positions with a high-confidence ancestral allele and of the remaining positions on the autosomes was done using the snpeff/5.0e tool [33] to see if any of these probability categories were specific to a region on the genome and no notable differences were observed in the proportions of the positions annotated and also in their consequences between the groups (see Additional file 5: Table S9).

**Table 2:** Number of ancestral allele calls (percentage) mapping to the major and minor alleles on the autosomes and X chromosome over the 1000 Bull Genomes, *Bos taurus* and *Bos indicus*

Group (N)	Ancestral allele call	Number of autosomal SNPs	Number of X chromosomal SNPs	Number of high confidence* autosomal SNPs	Number of high confidence* X chromosomal SNPs
Cattle (6191)	Major	59,296,006 (87.50)	2,090,158 (88.73)	55,007,099 (88.74)	1,765,733 (90.30)
	Minor	8,469,172 (12.50)	239,091 (10.26)	6,979,348 (11.26)	189,518 (9.69)
	Total	67,766,018	232,9275	61,987,061	1,955,267
*Bos taurus* (5204)	Major	59,122,728 (87.24)	2,085,396 (89.53)	54,842,130 (88.48)	1,761,321 (90.08)
	Minor	8,642,431 (12.75)	243,843 (10.47)	7,144,313 (11.52)	193,930 (9.92)
*Bos indicus* (606)	Major	60,338,000 (89.04)	2,128,601 (91.39)	56,039,349 (90.41)	1,814,511 (92.80)
	Minor	7,412,619 (10.94)	200,194 (8.60)	5,934,888 (9.57)	140,448 (7.18)

N: Number of samples; *high confidence reflects LR ≥ 0.8 and LR estimate supported by six or more out-group species

### Concordance of the ancestral alleles with previous studies

**Fig. 4:**
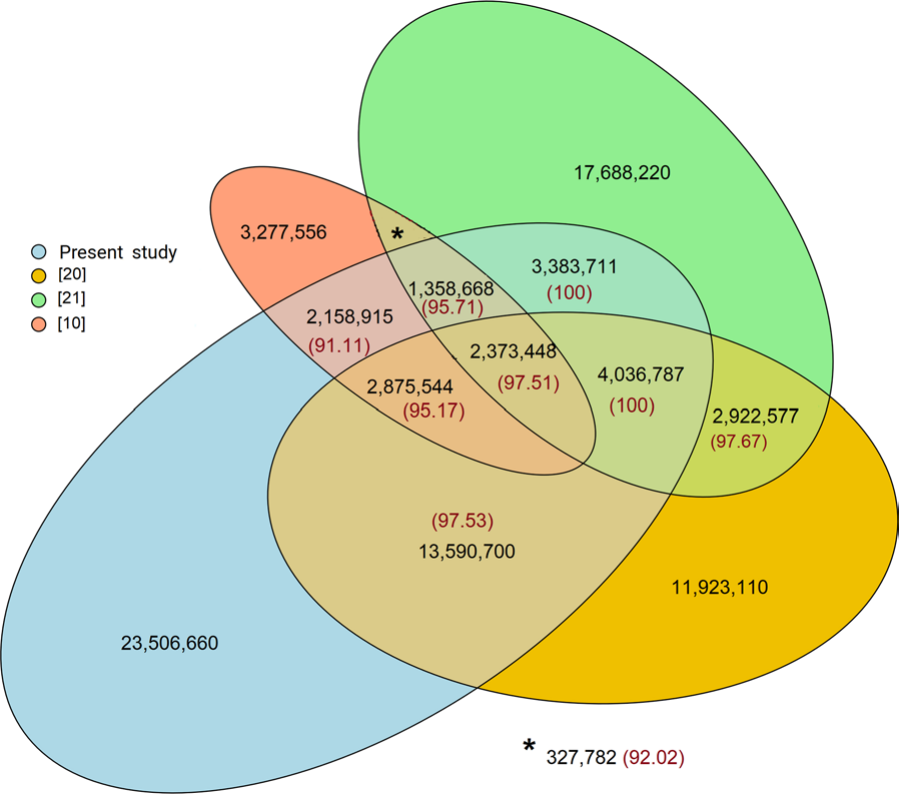
Coincidence of ancestral allele positions and ancestral alleles (percentage) among the studies for ancestral alleles with high confidence sites, i.e., ancestral alleles with a probability ≥ 0.8 and observed in at least six of the seven out-species included in the present study, and with a probability ≥ 0.8 in Xiang et al. [20]. Rocha et al. [10] and Naji et al. [21] do not provide probability estimates/confidence level of ancestral alleles and thus the whole list from these studies was used

Comparing ancestral alleles from the current study to those from three previous studies [10, 20, 21] showed a very high concordance. The ancestral alleles were highly concordant among the studies with a weighted average of 97.6% ranging from 91.1 to 100% between studies (Fig. 4), which validated 29.8 million ancestral alleles from the present study and another 3.2 million from the other studies, thus reaching a total of 33 million positions.

**Fig. 5:**
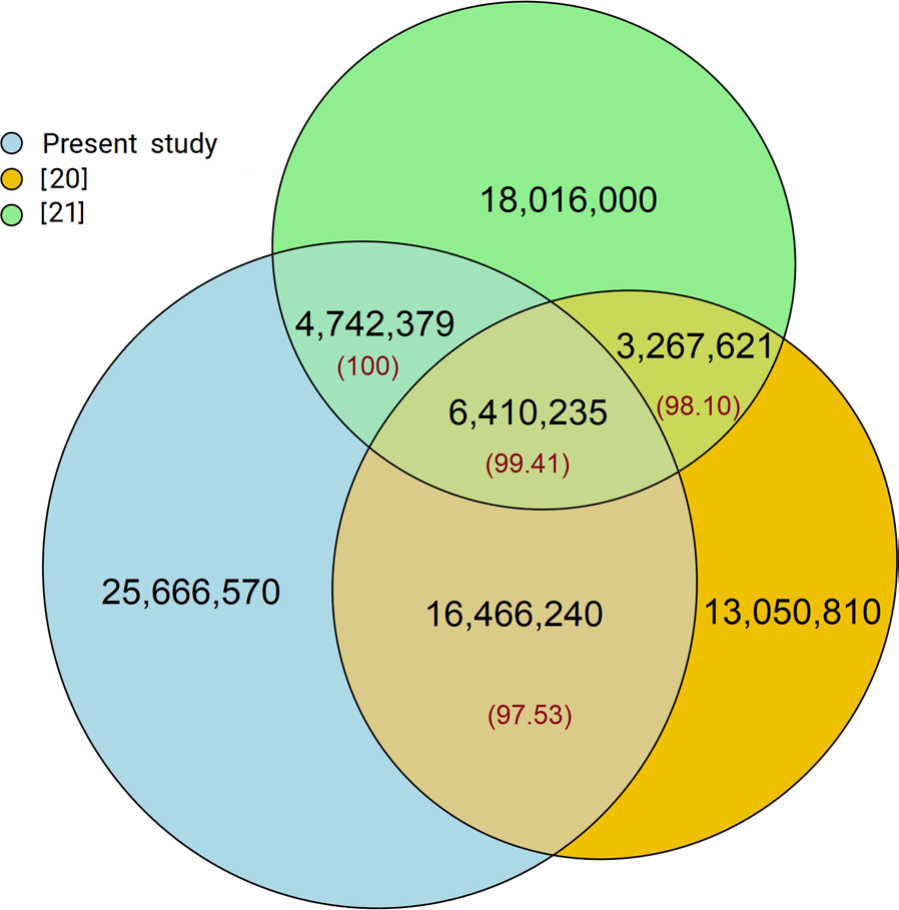
Extract of the coherence of ancestral allele positions and coincidence of ancestral alleles (percentage) among the studies based on the ARS-UCD1.2 reference genome considering ancestral alleles with high confidence sites, i.e., ancestral alleles with a probability ≥ 0.8 and observed in at least six of the seven out-species included in the present study, and with a probability ≥ 0.8 in Xiang et al. [20] from Fig. 4. Naji et al. [21] do not provide the confidence level of ancestral alleles and thus the whole set of ancestral alleles in the list was used

Similarly, the concordance of the ancestral alleles from this study with those from the two recent sequence-based studies of Naji et al. [21] and Xiang et al. [20] was high (98.4% in 27.6 million common positions) (Fig. 5). As such, there was a 99.4% concordance for 6.4 million positions shared by all three studies. As expected, ancestral alleles from the Xiang et al. [20] study and the present study shared more than 22.9 million positions with about 22.4 million ancestral alleles in concordance. Similarly, high concordance between the present study and that of Naji et al. [21] was found for 11.2 million positions. The coincidence between the Xiang et al. [20] and Naji et al. [21] studies was also equally high (> 99.0%).

For the X chromosome, among the previous studies only that of Xiang et al. [20] had a substantial number of ancestral alleles determined (843,609 positions) for the validation analysis. The number of high-confidence ancestral alleles in Xiang et al. [20] (probability > 0.8) was 803,184 and shared 571,807 genomic positions with the present study. The coincidence of ancestral alleles on the X chromosome between the two studies was 84.8% (i.e., 484,997 positions).

## Discussion

Our study makes available the largest list of ancestral alleles to date determined based on a LRT and using pre-identified variant positions in cattle by comparing with the maximum number of non-cattle *Bos* species sequences available. The present study alone presents 29.8 million autosomal ancestral alleles that are concordant with previous studies and another 23.5 million autosomal ancestral alleles of high confidence that are specific to this study. In addition, we identified 2.0 million high-confidence ancestral alleles for the X chromosome. We also demonstrated the use of our LRT to determine ancestral alleles with high accuracy.

The PC, which separated the out-species from the cattle group and with a clear delineation of the *Bos taurus* and *Bos indicus* species and provided sub-groupings within the out-species group, was as expected. However, the distribution of the species within the first PC was slightly different from that reported by Naji et al*.* [21]. This may be partly attributed to the alignment of the out-species sequences to the cattle reference genome and the use of selective variant positions (e.g., biallelic, high-confidence SNPs) from cattle to call genotypes, thereby substantially losing informative variants about the diversity of the out-species. Thus, the population structure observed in the current study is relative to a subset of variant positions in cattle.

Generally, the clustering within the out-species group (leaving out cattle) largely agreed with most of the previous studies although these are often based on mitochondrial sequences. For example, *B. frontalis* and *B. gaurus* were grouped together; as were *Bison bison* and *B. grunniens* [28, 35–37]. The clustering of *B. mutus* (wild yak) with *B. grunniens* (domestic yak) was expected based on the belief that domestic yak descended from the wild yak. Furthermore, the placement of *B. sauveli* closer to the gayal and gaur group in this study was in line with several previous studies [24, 29]. *B. javanicus* has been consistently placed among the *B. gaurus* and *B. frontalis* group in previous studies based on mitochondrial sequences [35] but, in our study, it was placed separately from these species. However, the use of such closely-related species as separate groups (e.g., domestic and wild yaks) can potentially bias the ancestral allele call, particularly when the ancestral allele is only supported by two close groups. These variations in the placement of species could be attributed to substantial differences in the number of variants considered between studies. Altogether, the PCA structure largely corroborated previously reported results and suggested that the data processing underpinning our study is solid. As such, PCA-based ancestral allele determination is currently applied in practice [21] and the genetic distance between the out-species and cattle may be considered to draw confidence statistics.

The ability of our study to determine, validate and confirm a high coherence of the determined ancestral alleles with those from previous studies suggests two key points. First, our approach that uses pre-determined variant positions for the identification of ancestral alleles to generate a larger set of ancestral alleles is effective. In other approaches, the detection of variants depends on the sampling size and the diversity of the samples, which is often restricted in smaller studies. Our approach may also be used in other species for which sequence-level genotype data are available. Second, regardless of the approaches used for the determination of ancestral alleles, in general, the agreement of ancestral allele calls across studies remained very high (~ 97%). This implies that the specific ancestral alleles determined in the previous studies and in the current study are also of equally high confidence for further analyses. However, some false positives might be present due to the use of similar approaches in different studies, reference biases that arise from the alignment of out-species to the cattle reference genome and the use of conservative filtering of read depth and quality. Other potential biases and limitations, particularly for the X chromosome, can arise from not taking the sex and hemizygosity of the samples into account in the determination analyses, and warrant further investigation.

The combination of ancestral alleles across studies further enhances the coverage of ancestral alleles on the genome. While Xiang et al. [20] used a subset of the data from the 1000 Bull Genomes project, they presented about 11.9 million ancestral alleles specific to their study, with 5.2 million being outside of the filter and thresholds considered in our study. Similarly, from the 32.4 million position explored in Naji et al. [21], 17.7 million were unique to their study, with 13.2 million distinct from the 1000 Bull Genomes project (see Additional file 5: Table S10). Thus, a combination of our study with the three previous studies is assumed to result in validated ancestral alleles for 32.7 million genomic positions and about 56.4 million study-specific ancestral alleles to substantially increase the current list of reliable ancestral alleles in cattle to over 90 million.

Unlike the global use of ancestral alleles or ancestral sequences in the study of evolutionary history and the origin of species, the objective of the ancestral alleles determined in our study is to use them to decipher their association with production traits and signatures of selection in modern cattle, i.e. in other words, to identify ancestral alleles from the variants identified in cattle that are widely used for genome imputation and association testing to understand the effect of ancestral alleles on traits of economic importance in cattle. Thereby, to selectively use known biallelic variants in cattle determined by aligning short read sequences to the cattle reference genome and variant calling. The same approach has been used to call variants in out-species at the identified positions aligning to a cattle reference genome. Variant or SNP-based ancestral allele call has been used in cattle and humans in previous studies [10, 20, 21, 38]. While reference-based alignment has shortcomings, including reference biases [39], it enables the use of short read sequences of multiple samples per species from next-generation sequencing platforms to capture the allelic diversity at a position both within and across the out-group species, which is key for defining ancestral alleles in our approach, unlike the multiple sequence alignment (MSA) approach where a single reference genome sequence per species is used. The reference-free alignment MSA approaches (e.g., Cactus, Enredo, Pecan) not only overcome the reference biases but also consider the insertions, deletions, substitutions and copy number variations among the species [40–42] and also allows identification of conserved sequence patterns and motifs in a whole-sequence family that are an essential prerequisite for phylogenetic analysis [43]. However, overemphasis sometimes produced ancestral sequences that are longer than the true sequences. The differences in the algorithm of MSA affect the accuracy of the determination of ancestral alleles, and the appropriate choice is critical [44].

Our approach that uses an LRT based on genotype frequencies to assign ancestrality is equivalent to the est-sfs approach of Keighley and Jackson [23] but has a subtle difference. The est-sfs algorithm operates at the level of the nucleotide frequencies and, in its current implementation (https://sourceforge.net/projects/est-usfs/), handles a maximum of up to three outgroups. Instead, our approach operates at the level of the genotype frequencies for bi-allelic SNPs only, but there are no limits to the number of outgroups. Ancestral alleles have also been determined within human populations without references to out-group species using haplotype diversities and led to the identification of reliable variants that are recent [38].

The strikingly high concordance of the ancestral alleles determined here with those from previous studies suggests that the LRT is a satisfactory tool to determine ancestral alleles and probability estimates for ancestrality. While it would have been interesting to compare ancestral alleles using our approach with other MSA-based approaches, it was beyond the scope of the current study but warrants future investigation on the accuracy and scalability to determine whole ancestral reference genome. Currently, because of its computational efficiency, coupled with the need to organise the data in populations, we believe that the LRT proposed to assign ancestrality can be easily implemented for the identification of signatures of selections. For instance, the dataset could be split into cattle populations on one side and out-species on the other. Then, the application of the LRT, separately, in each population would identify alleles that change “ancestrality” assignment and the genomic regions where these alleles are mapped to and mined for signatures of selection.

Overall, considering the general definition of ancestral alleles as the allelic state of the last common ancestors, the last common ancestor as the upper bound can be more recent within a family, across families or beyond which are specific to the scope of the study. Thus, an approach such as ours that uses within-population variation may be more suitable to study more recent evolutionary events compared to the conventional approaches that can draw inferences over a large spectrum of unrelated species for a longer molecular evolutionary history.

## Conclusions

Our findings suggest that ancestral alleles can be determined from the predetermined variants in a species. They highlight the high concordance of the ancestral alleles determined across studies, in spite of the differences in the approaches used for their determination. They also demonstrate that a simple test, such as the likelihood ratio test, can be used as an alternate statistical tool to determine ancestral alleles with high accuracy.

### Supplementary Information

**Additional file 1: Table S1.** Variant categories by confidence and number of variants in the1000 Bull Genomes project (Run9). **Table S2.** Accession Number and Project ID of the out-species samples used in this study for the determination of ancestral alleles including the cattle group used for principal component analysis. **Table S3.** Attribution for the images used in Fig. 1.

**Additional file 2: Method S1.** Tools, command lines and thresholds used in processing of raw reads (fastq) to gVCF.

**Additional file 3: Method S2.** Steps for determining ancestral alleles using the likelihood ratio test. **Table S4.** Defining genotype configuration from genotype frequency, signal allocation, estimation likelihood ratios for alleles. **Table S5.** Likelihood ratio assignment (LR_a_; last two columns) for each site per the GT_c_ from Table S3. **Table S6.** Putative ancestral allele and probability of ancestrality for alleles. **Table S7.** Species support for ancestral alleles by the number of species called at a site. The sites highlighted in bold demonstrate how species contribute to accessing ancestrality probability.

**Additional file 4: Method S3.** Bash script for determining ancestral alleles using the likelihood ratio test steps 1 to 3 in Additional file 3: Method S2.

**Additional file 5: Table S8.** Summary of likelihood ratios for major and minor alleles designated as ancestral alleles and number of species contributing to the likelihood ratio. **Table S9.** Annotation of variant positions of ancestral alleles from high confidence and low/failed categories. **Table S10.** Partitioning of positions of ancestral alleles according to categories in the1000 Bull Genomes project.

## Data Availability

The variant positions and corresponding alleles used for determining ancestral alleles in this study are available from the 1000 Bull Genomes project on request. The raw sequence data of the samples used in this study are available from NCBI (see Additional file 1: Table S2). The pipeline including scripts for determining ancestral alleles in this study is available as supplementary information (see Additional file 3: Method S2 and Additional file 4: Methods S3). The ancestral alleles derived in this study are available from CSIRO’s data access portal at https://doi.org/10.25919/9a81-4p83.
