# Supplementary material for: Ancestral alleles defined for 70 million cattle variants using a population-based likelihood ratio test
Source: Genet Sel Evol. 2024 Feb 6;56:11. doi: 10.1186/s12711-024-00879-6 (PMC10848479; doi:10.1186/s12711-024-00879-6)
Supplement: Supplementary file 3 — Additional file 3: Method S2. Steps for determining ancestral alleles using the likelihood ratio test. Table S4. Defining genotype configuration from genotype frequency, signal allocation, estimation likelihood ratios for alleles. Table S5. Likelihood ratio assignment (LRa; last two columns) for each site per the GTc from Table S3. Table S6. Putative ancestral allele and probability of ancestrality for alleles. Table S7. Species support for ancestral alleles by the number of species called at a site. The sites highlighted in bold demonstrate how species contribute to accessing ancestrality probability. [file 12711_2024_879_MOESM3_ESM.docx]

**Ancestral alleles defined for 70 million cattle variants using a population-based likelihood ratio test**

Jigme Dorji, Antonio Reverter, Pamela A Alexandre, Amanda J Chamberlain, Christy J Vander-Jagt, James Kijas and Laercio R Porto-Neto

**Additional file 3 Method S2: Steps for determining ancestral alleles using the likelihood ratio test**

1. ***Determination of ancestral alleles***
2. ***Weighting the ancestral allele with the number of species supporting the call***
3. ***Determination of ancestral alleles using likelihood ratio***

The genotypes of the animals within each out-species are extracted from the VCF and the genotype frequency (GT_f_) at each genomic site is determined by counting the number of animals that are homozygous REF (0/0), heterozygous (0/1 or 1/0) and homozygous ALT (1/1) for that site. Then the GT_f_ for all genomic sites within a species are summarized to determine the total number of unique GT_f_ configurations (GT_c_) across the three possible genotypes, and the number of sites where a given GT_c_ is observed (GT_c_obs_).

By chance, the expected number of sites per unique GT_c_ (GT_c_exp_) is the ratio between the total number of sites considered and the number of unique GT_c_. Hence, the likelihood ratio (LR) of one of the alleles to be ancestral can be defined as GT_c_obs_ divided by GT_c_exp_:

$likelihood ratio=LR= \frac{{GT}_{c\_obs}}{{GT}_{c\_exp}}$.

Then, the LR value is assigned to the allele corresponding to the genotype with the largest number of animals and indicates the signal for ancestrality. That is, if the largest number of animals are (0/0) then the ancestrality signal goes to A_1_ (first allele) and the LR value is assigned (LR_a_) to A_1_ (LR_a_*A_1_*) while a 0 is assigned to A_2_ (second allele). Instead, if the highest number of animals are (1/1) then the ancestrality signal goes to A_2_ and the LR value is assigned to A_2_ (LR_a_*A_2_*) while a 0 is assigned to A_1_. However, in cases where the number of animals is equal for both homozygous genotypes, the signal is not assigned and recorded as 0 for both LR_a_*A_1_* and LR_a_*A_2_*. Similarly, heterozygous genotypes provide no signal towards allele ancestrality. For instance, consider a situation with 1000 sites and 10 animals of a given out-species and let the observed GT_f_ be such that only 5 distinct GT_c_ (1 to 5) are observed as follows:

Additional file 3 Table S4. Defining genotype configuration from genotype frequency, signal allocation, estimation likelihood ratios for alleles

| **GT_c_** | **GT_f_** | | | **Signal goes to** | **GT_c_obs_** | **GT_c_exp_** | **LR** | **LR**_a_ | |
| --- | --- | --- | --- | --- | --- | --- | --- | --- | --- |
|  | (A_1_A_1_) | (A_1_A_2_) | (A_2_A_2_) |  |  |  |  | *A_1_* | *A_2_* |
| 1 | 9 | 1 | 0 | A_1_ | 400 | 200 | 2.00 | 2.00 | 0 |
| 2 | 9 | 0 | 1 | A_1_ | 200 | 200 | 1.00 | 1.00 | 0 |
| 3 | 5 | 0 | 5 | None | 50 | 200 | 0.25 | 0 | 0 |
| 4 | 2 | 6 | 2 | None | 50 | 200 | 0.25 | 0 | 0 |
| 5 | 0 | 1 | 9 | A_2_ | 300 | 200 | 1.50 | 0 | 1.5 |

In the above example, the first configuration (GT_c_ = 1) is such that 9 animals have genotype (0/0), 1 animal has genotype (0/1) or (1/0), and no animal has genotype (1/1), and this configuration happens in GT_c_obs_ = 400 sites. The five unique GT_c_ across the 1000 sites range in GT_c_obs_ from 50 to 400, while GT_c_exp_ by chance alone is 200 for each configuration (from 1000/5). The first two configurations (GT_c_ 1 and 2) signal towards the first allele (A_1_) as the ancestral while the third fourth configuration (GT_c_ 3 and 4) does not signal either allele. The last configuration signals the second allele (A_2_) as the ancestral. However, the LR indicates that the first and last configurations are more frequent than by chance alone, while the second is no different than chance and configurations third and fourth are less likely than chance alone.

Then, using Additional file 3 Table S5 as a reference, each site in the genome gets a LR_a_ as follows:

Additional file 3 Table S5. Likelihood ratio assignment (LR_a_; last two columns) for each site per GT_c_ from Additional file 3 Table S4

| **Site** | **Chr** | **Pos (BP)** | **A_1_** | **A_2_** | **GT_f_** | | | **GT_c_** | **LR_a_** | |
| --- | --- | --- | --- | --- | --- | --- | --- | --- | --- | --- |
|  |  |  |  |  | *A_1_A_1_* | *A_1_A_2_* | *A_2_A_2_* |  | *A_1_* | *A_2_* |
| 1 | 1 | 28045 | G | T | 9 | 0 | 1 | 2 | 1.00 | 0 |
| 2 | 1 | 29810 | G | T | 5 | 0 | 5 | 3 | 0.25 | 0 |
| 3 | 1 | 37566 | G | A | 2 | 6 | 2 | 4 | 0 | 0 |
| 4 | 1 | 40738 | A | T | 9 | 1 | 0 | 1 | 2.00 | 0 |
| 5 | 1 | 41701 | A | T | 9 | 0 | 1 | 2 | 1.00 | 0 |
| 6 | 2 | 37249 | A | C | 2 | 6 | 2 | 4 | 0 | 0 |
| 7 | 2 | 54197 | C | G | 9 | 1 | 0 | 1 | 2.00 | 0 |
| 8 | 2 | 74761 | A | C | 0 | 1 | 9 | 5 | 0 | 1.50 |
| 9 | 2 | 86009 | C | T | 9 | 0 | 1 | 2 | 1.00 | 0 |
| 10 | 2 | 88161 | G | A | 9 | 0 | 1 | 2 | 1.00 | 0 |
| 11 | 3 | 19593 | A | C | 9 | 1 | 0 | 1 | 2.00 | 0 |
| 12 | 3 | 25445 | T | C | 2 | 6 | 2 | 4 | 0 | 0 |
| 13 | 3 | 27448 | C | A | 0 | 1 | 9 | 5 | 0 | 1.50 |
| 14 | 3 | 33835 | T | C | 9 | 0 | 1 | 2 | 1.00 | 0 |
| 15 | 3 | 34512 | A | G | 9 | 1 | 0 | 1 | 2.00 | 0 |
| … | … | … | … | … | … | … | … | … | … | … |
| 1000 | 29 | 51098474 | A | G | 5 | 0 | 5 | 3 | 0 | 0 |

The above procedure is repeated to generate the above two tables for all the out-species. Note that while the genomic sites are the same for all species (i.e., 1000 in our toy example), the number of genotype configurations (GT_c_) and hence LR_a_*A_1_* and LR_a_*A_2_* are likely vary for each out-species. Once all out-species have been processed, signals LR_a_*A_1_* and LR_a_*A_2_* at a position are summed across the species to get the global signals as G${LR}_{a}$(A_1_) and G${LR}_{a}$(A_2_) for either allele at each position as follows:

${G{LR}_{a}(A}_{1})=\sum_{k=1}^{n} {{LR}_{a}{(A}_{1})}_{spp(k)}$,

${G{LR}_{a}(A}_{2})=\sum_{k=1}^{n} {{LR}_{a}{(A}_{2})}_{spp(k)}$.

The ancestrality of allele at a position is determined as the allele (A_1_ or A_2_) corresponding to the genotype with highest *GLR_a_* signal (Additional file 3 Table S6). The probability of A1 and A2 being the ancestral allele at a site is calculated as the ratio of their across-species signal to the sum of both global signals:

${P(A}_{1})=\frac{{G{LR}_{a}(A}_{1})}{{G{LR}_{a}(A}_{1})+{G{LR}_{a}(A}_{2})}$,

${P(A}_{2})=\frac{{G{LR}_{a}(A}_{2})}{{G{LR}_{a}(A}_{1})+{G{LR}_{a}(A}_{2})}$.

Then, the probabilities of each allele to be ancestral at all the position can summarized (Additional file 3 Table S6).

Additional file 3 Table S6. Putative ancestral allele and probability of ancestrality for alleles

| **Site** | **Chr** | **BP pos** | **A_1_** | **A_2_** | **G**$\boldsymbol{LR}_{\boldsymbol{a}}$ | | **Ancestral allele** | **Prob** | |
| --- | --- | --- | --- | --- | --- | --- | --- | --- | --- |
|  |  |  |  |  | A_1_ | A_2_ |  | A_1_ | A_2_ |
| 1 | 1 | 28045 | G | T | 6.00 | 0 | G | 1 | 0 |
| 2 | 1 | 29810 | G | T | 3.95 | 0 | G | 1 | 0 |
| 3 | 1 | 37566 | G | A | 0 | 0.80 | A | 0 | 1 |
| 4 | 1 | 40738 | A | T | 15.00 | 0 | A | 1 | 0 |
| 5 | 1 | 41701 | A | T | 6.00 | 0 | A | 1 | 0 |
| 6 | 2 | 37249 | A | C | 0.50 | 0 | A | 1 | 0 |
| 7 | 2 | 54197 | C | G | 6.00 | 0 | A | 1 | 0 |
| 8 | 2 | 74761 | A | C | 1.00 | 1.80 | C | 0.36 | 0.64 |
| 9 | 2 | 86009 | C | T | 4.00 | 3.00 | C | 0.57 | 0.43 |
| 10 | 2 | 88161 | G | A | 6.00 | 0 | G | 1 | 0 |
| 11 | 3 | 19593 | A | C | 4.50 | 0.60 | A | 0.88 | 0.12 |
| 12 | 3 | 25445 | T | C | 2.00 | 0 | T | 1 | 0 |
| 13 | 3 | 27448 | C | A | 1.90 | 1.50 | C | 0.56 | 0.44 |
| 14 | 3 | 33835 | T | C | 4.00 | 4.00 | - | 0 | 0 |
| 15 | 3 | 34512 | A | G | 2.00 | 2.80 | G | 0.42 | 0.58 |
| … | … | … | … | … | … | … | … | … | … |
| 1000 | 29 | 51098474 | A | G | 3.95 | 0 | G | 1 | 0 |

1. ***Weighting the ancestral allele with the number of out-species evidence support***

Because some sites might be missing in a given out-species (i.e., with all animals in that out-species having a missing genotype for those sites), the number of out-species for which a given site is observed can be used as additional evidence supporting the ancestrality probability computed in the previous steps. The strength of this support can be ascertained from the cumulative number of sites with one to “*n*” number of out-species observed, where “*n*” is the total number of out-species under consideration.

In our toy example of 1000 positions, if 250 sites were supported by one, 600 sites by two and 150 sites by three out-species, it would mean a cumulative of 250 sites for one species, 850 for two species and 1000 for three species. Accordingly, the support for 1 to 3 species can be assigned as 250, 850 and 1000 divided by 1000 respectively as in Additional file 3 Table S7.

Additional file 3 Table S7. Species support for ancestral allele by the number of species called at a site. The sites highlighted in bold demonstrate the how species support contribute to accessing ancestrality probability

| **Site** | **Chr** | **Pos (bp)** | **A_1_** | **A_2_** | $\boldsymbol{G}\boldsymbol{LR}_{\boldsymbol{a}}$ | | **Ancestral allele** | ***Prob*** | | **No. of out-Spp called** | **Out-spp support** |
| --- | --- | --- | --- | --- | --- | --- | --- | --- | --- | --- | --- |
|  |  |  |  |  | A_1_ | A_2_ |  | A_1_ | A_2_ |  |  |
| 1 | 1 | 28045 | G | T | 6.00 | 0 | G | 1.00 | 0 | 3 | 1.00 |
| 2 | 1 | 29810 | G | T | 3.95 | 0 | G | 1.00 | 0 | 3 | 1.00 |
| 3 | 1 | 37566 | G | A | 0 | 0.80 | A | 0 | 1.00 | 3 | 1.00 |
| 4 | 1 | 40738 | A | T | 15.00 | 0 | A | 1.00 | 0 | 3 | 1.00 |
| 5 | 1 | 41701 | A | T | 6.00 | 0 | A | 1.00 | 0 | 2 | 0.85 |
| 6 | 2 | 37249 | A | C | 0.50 | 0 | A | 1.00 | 0 | 2 | 0.85 |
| 7 | 2 | 54197 | C | G | 6.00 | 0 | A | 1.00 | 0 | 2 | 0.85 |
| 8 | 2 | 74761 | A | C | 1.00 | 1.80 | C | 0.36 | 0.64 | 1 | 0.25 |
| **9** | **2** | **86009** | **C** | **T** | **4.00** | **3.00** | **C** | **0.57** | **0.43** | **1** | **0.25** |
| 10 | 2 | 88161 | G | A | 6.00 | 0 | G | 1.00 | 0 | 2 | 0.25 |
| 11 | 3 | 19593 | A | C | 4.50 | 0.60 | A | 0.88 | 0.12 | 2 | 0.85 |
| 12 | 3 | 25445 | T | C | 2.00 | 0 | T | 1.00 | 0 | 2 | 0.85 |
| **13** | **3** | **27448** | **C** | **A** | **1.90** | **1.50** | **C** | **0.56** | **0.44** | **3** | **1.00** |
| 14 | 3 | 33835 | T | C | 4.00 | 4.00 | - | 0 | 0 | 1 | 0.25 |
| 15 | 3 | 34512 | A | G | 2.00 | 2.80 | G | 0.42 | 0.58 | 1 | 0.25 |
| … | … | … | … | … | … | … | … | … | … | … | … |
| 1000 | 29 | 51098474 | A | G | 3.95 | 0 | G | 1.00 | 0 | 3 | 1.00 |

For instance, in the above example the site on Chr 3 27448 bp for a 0.56/0.44 ancestral probabilities and with three out-species support is more reliable than the very similar probabilities of 0.57/0.43 from the site on Chr 2 86009 bp but with only 1 out-species support.
